# Primary or revision arthroplasty with an integrated acetabular cup—MUTARS^®^ RS cup system

**DOI:** 10.1007/s00068-022-01956-5

**Published:** 2022-03-31

**Authors:** Maren Janko, René Verboket, Maria Genari, Johannes Frank, Ingo Marzi

**Affiliations:** Department of Trauma-, Hand- and Reconstructive Surgery, Goethe University Frankfurt, University Hospital, Frankfurt am Main, Germany

**Keywords:** MUTARS^®^ RS cup system, Hip revision, Intraoperative procedure, Feasibility

## Abstract

**Introduction:**

The aim of this article is to show a new concept of indication and application of the MUTARS^®^ RS Cup System in primary and revision hip arthroplasty. This integrated system is applicable for different acetabular cup replacements in patients with acetabular fractures or instable defects, as well as periprosthetic acetabular fractures. The MUTARS^®^ RS Cup System is a cementless revision cup for insertion into the acetabulum with an integrated polyethylene cup, which fits to a regular or bipolar head. This system replaces the conventional approach for acetabular revision with a Burch-Schneider ring, in which a normal polyethylene cup is cemented. This interface with its complications is avoided with this system of a titanium revision cup with integrated polyethylene cup. Steps of preoperative planning and the intraoperative implementation will be highlighted in this article.

**Material and methods:**

This system was applied in 49 patients with 52 MUTARS^®^ RS Cup Implantations in 30 males, 22 females, with an average age of 76,1 years (36,9–94,4 years).

**Results and discussion:**

The system shows a good operative feasibility, as well as a reliable handling and safe method for stable treatment of non-reconstructable acetabular fractures or acetabular incongruencies and instabilities.

## Introduction

The aging population and the increasing prevalence of obesity will continue to increase the number of hip replacements. Although there are good clinical results, especially regarding the lifetime of prostheses, many patients outlive the typical lifetime of implants and a high number of prosthesis changes are necessary. Revision is only necessary in a minority of cases (about 17% of hip prostheses fail), but when prosthesis failure occurs, a significant number of acetabular cup failures occurs [1]. Revisions of peri-implant and periprosthetic acetabular fractures, usually with bone defects and/or reduced bone quality, increase due to the aging society and present a technical challenge to the surgeon [2–5]. Therefore, we present and discuss the advantages and disadvantages of the procurable MUTARS^®^ RS cup system, as well as the clinical indication for acetabular cup revision, radiographic classification systems, and preoperative planning.

Typically, patients present hip pain after trauma or even without trauma. A comprehensive anamnesis and specific physical examination should be performed on all patients regardless of the clinical presentation. For preoperative planning, an anteroposterior (AP) pelvic X-ray with planning ball is necessary. Often, a 3D-CT scan in a thin layer technique of the pelvis is also performed, which shows the defect of the acetabulum.

### Bone defect assessment

The development of a reliable, valid, and generally accepted classification for acetabular bone loss during revision of total hip arthroplasty (THA) is still problematic. Despite its limitations, the Paprosky classification has many advantages, including its widespread familiarity, ease of use, availability of routine perioperative radiographs and adequate reliability and validity [6]. Moreover, given that the Paprosky classification can be used to predict implant demand, this system is being used in an increasing number of studies to report medium- and long-term results for revision of THA with acetabular bone loss [7–9]. The Paprosky classification is one of the best available options to assist surgeons in anticipating and planning for findings at the time of revision surgery [6]. In addition, a study has shown that the reliability of classification has been increased by dedicated teaching [10], suggesting that classification could be more reliable if surgeons were specifically trained in its use [11]. The classification covers Type I (I) to Type IV (IV). The most severe type is Type IV with extensive metaphyseal and diaphyseal bone loss and unsupported isthmus. Since, as a rule, the defect intraoperatively is often found to be larger than expected, the existing defect classifications should only be regarded as a strategic tool.

### Indications for acetabular revisions

The indications for surgical therapy are revision surgery for a loose cup with defects in the acetabulum. Furthermore, periprosthetic acetabular fractures with residual instability are an indication for treatment. In general, acetabular fractures can also be bridged with the acetabular cup and tumor diseases in the area of the acetabulum, if not a complete instability of the pelvic ring is present [5, 12].

**Fig. 1:**
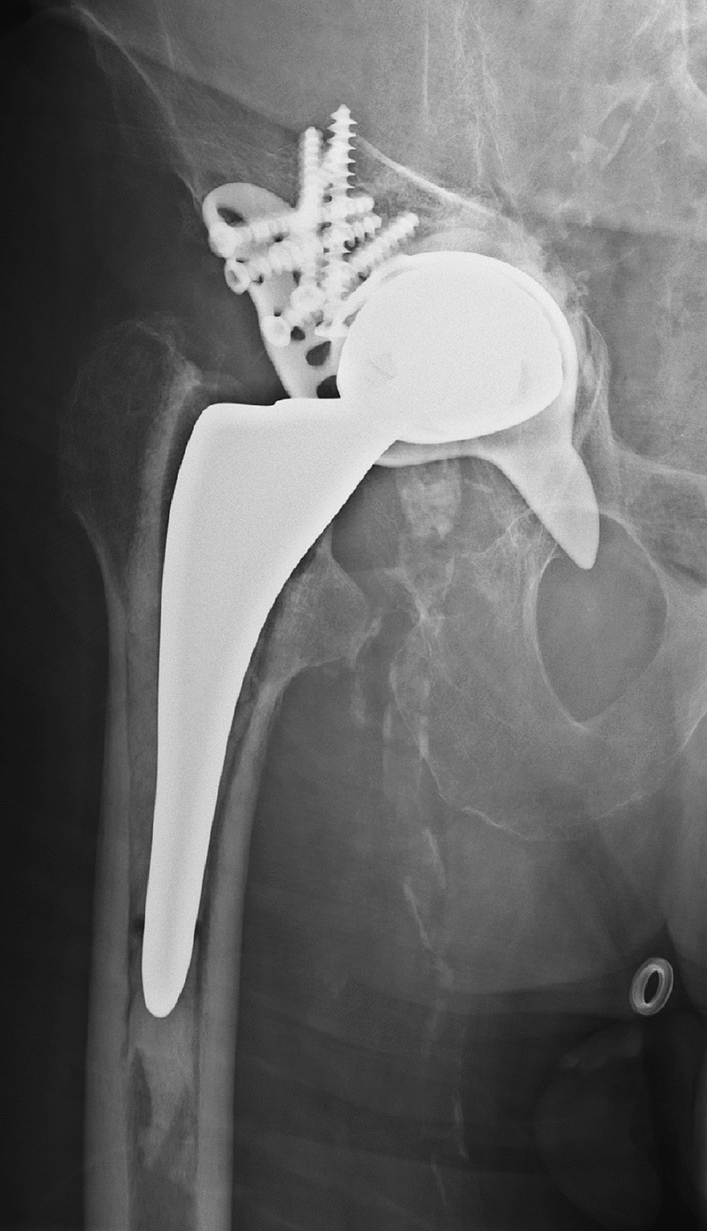
Shows this conventional approach with a Burch Schneider Ring and its complications with a break-out of the distal flag ring system into the small pelvis

**Fig. 2:**
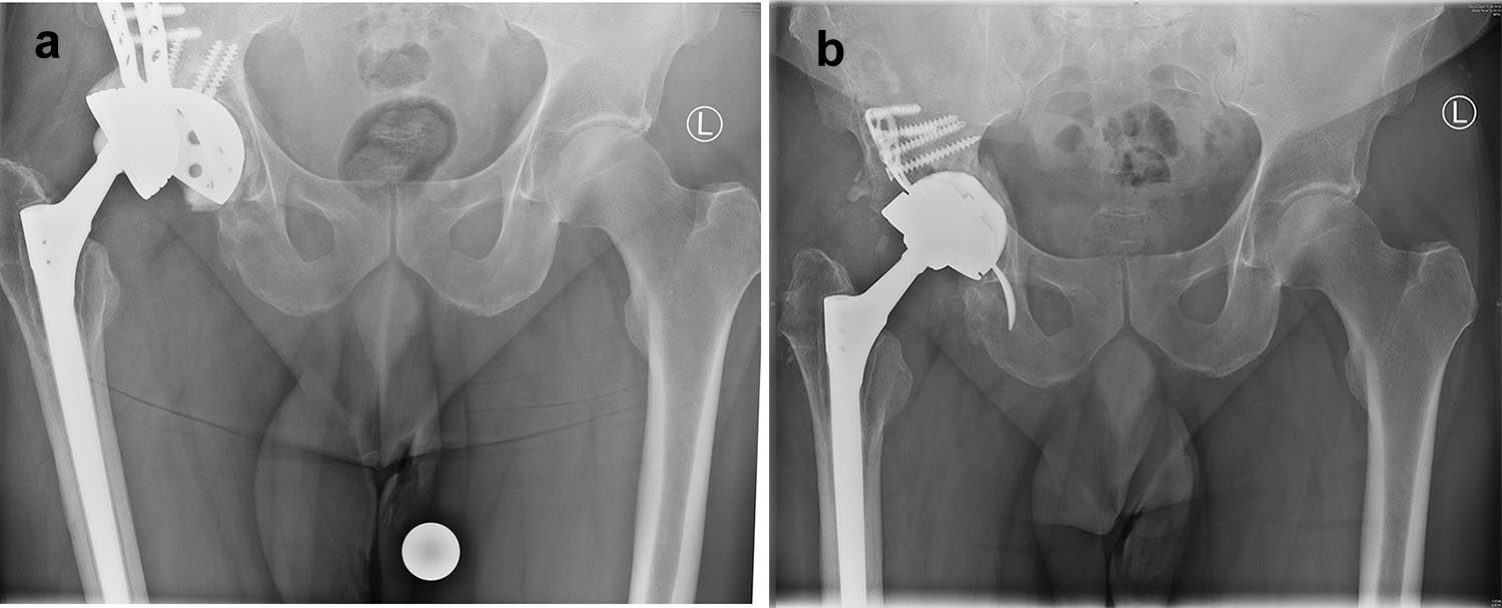
**a** Recurrent subluxations of the right extended revision TEP. **b** Replacement of the insertion of a MUTARS^®^ RS Cup prosthesis while remaining the femoral stem. Well-fitting cup and prosthesis in the pelvis overview

The common approach to date is the use of a Burch-Schneider ring, which is inserted in the ischium and screwed to the ilium [13, 14]. Following stabilization of this titanium ring, a regular polyethylene cup is cemented into the titanium cup with the requirement for a correct inclination and ante-version angle. Only 28- or 32 mm heads fit this low-profile polyethylene cup, so larger heads and tripolar heads are not available for these situations. This leads in particular after revision arthroplasty to cut outs or luxation, or even a break-out of the acetabular ring [14] (Fig. 1). An additional problem is the loosening of the cement fixation of the polyethylene cup over time [15] and revision arthroplasty is limited to cemented cups (Fig. 2a).

As an alternative, MUTARS^®^ revision operations of THA are an ideal system for complex cases, e.g., with acetabular cup loosening, peri-implant and periprosthetic fractures with protrusion of the prosthesis head into the acetabulum and pronounced bone defects in the area of the acetabulum. These defects can be caused by failure of the acetabular implant, which can happen through aseptic loosening, infection, instability, trauma and osteolysis as indicated above [16]. As a further advantage, also tripolar prosthesis can be included in this system (Fig. 2b).

In the following, we report on our large experience with this system at various complex indications. The MUTARS^®^ RS Cup offers a solution to the above problems and was introduced to the market in 2012. Until the end of 2018, 2,185 MUTARS^®^ RS Cups worldwide have been sold [17], but only very few reports have been published so far. During follow-up examinations, the MUTARS^®^ RS Cup showed clinically safe follow-ups and good applicability for appropriate indications [17].

## Methods

All patients received the MUTARS^®^ RS Cup System (implantcast GmbH, Buxtehude, Germany) between 03/2016 and 03/2021. The size of the MUTARS^®^ RS Cup System (available from size 46 mm to 62 mm, 5 sizes) is measured on a pre-operatively created image using a planning ball (MediCAD, Hectec GmbH, Atlanta, USA). The planning ball has to be placed at the same level as the hip joint to measure the standard size.

### OP procedure

**Fig. 3:**
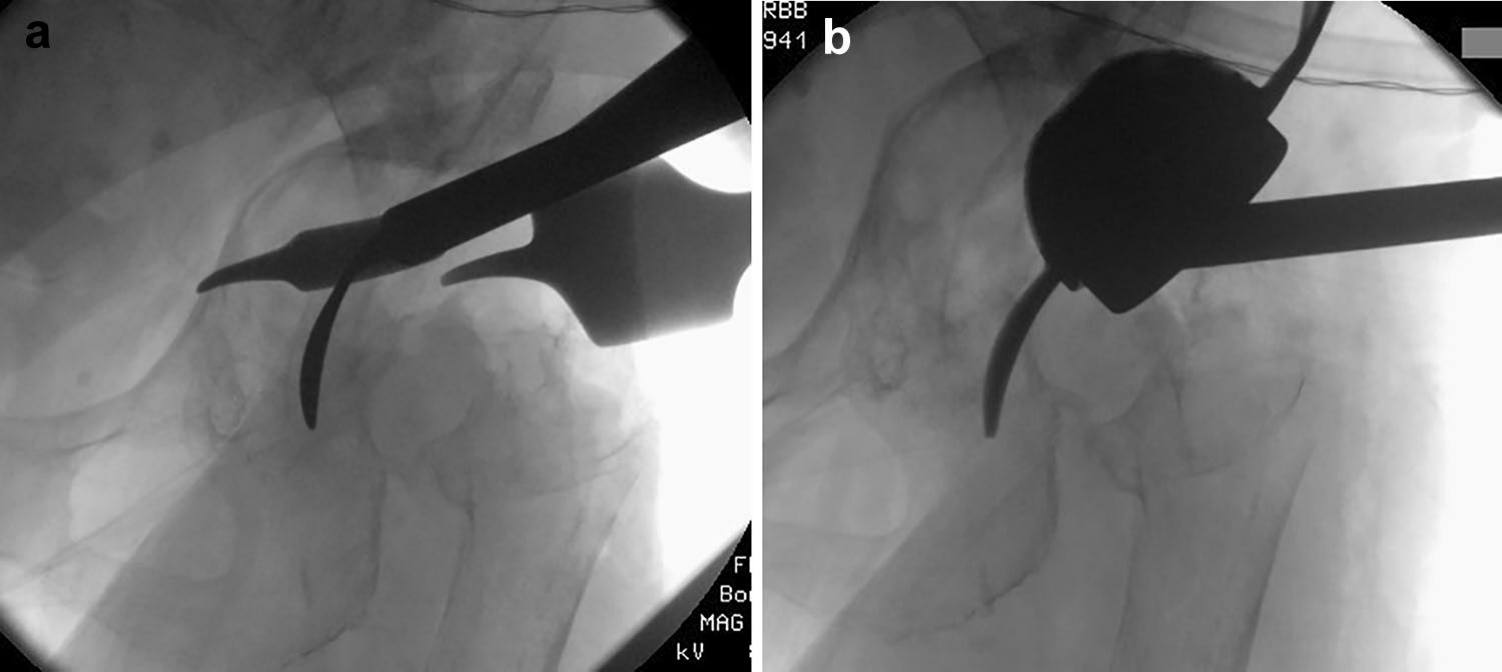
**a** It shows the left acetabulum after careful sequestrotomy and curettage with drill out to the desired size. After opening of the os ischii with the straight opening instrument, now shown the inserted curved instrument. **b** The MUTARS^®^ RS Cup System is inserted into the opened os ischii with the flap. The cup is not yet in the correct position and must still be knocked into the desired position

The usual anterolateral approach to the hip joint is performed. First of all, a good visualization of the acetabulum and a detailed sequestrotomy and curettage of the acetabular bone with assessment of the defect are necessary. This is followed by the preparation of the os ischii and the opening of the os ischii using the opening instruments. The first step is to insert the straight opening instrument atrium into the os ischii bone. Next, the same aperture is prepared with the curved second awl (Fig. 3a). The MUTARS^®^ prosthesis is placed on a special impactor that facilitates insertion. Under X-ray control, the MUTARS^®^ RS Cup System is attached in the appropriate size and inserted into the os ischii with the flap (Fig. 3b). It is important to avoid retroversion when hammering in. Now the cup system fits exactly into the acetabulum.

The cup is secured with two to seven screws in the area of the os ilium. The screws are inserted with spacers to prevent slipping through the ovals holes of the bracket. Again, care must be taken to ensure that the RS cup has a particular antetorsion around 20 degrees to avoid the dorsal dislocation tendency. An additional radiological check is recommended in at least two levels. Subsequently, the securing pin is inserted and the polyethylene inlay is placed with the elevation towards dorsocranial to additionally prevent dislocation. The corresponding head for the bipolar prosthesis is then fitted. After implantation of the femur stem, reposition of the hip joint is performed. Subsequently, the mobility of the hip joint is checked, as well as the rotation and leg length of the patient. The Lumic^®^ system, the option for a tripolar prosthesis, can be used in unstable conditions. This is a bipolar anchoring option that can be driven into the cup. Of the tripolar cups, there are the coupled cups and the uncoupled dual-mobility cups. The uncoupled version shows a better survival and revision rate. The indications for dual-mobility cups are recurrent dislocations and situations in which there is a higher risk of dislocation [18].

## Results

From March 2016 to March 2021, a total of 49 patients were included in the study, during which all of them received the MUTARS^®^ RS Cup. In total, the MUTARS^®^ RS Cup were installed 52 times, twice in 3 patients. The mean age of patients was 76,1 years (36,9–94,4 years), 28 male and 21 female patients. We included sequentially all patients in the study.

Indications for this intervention arrived from the preoperative radiological examinations. Acetabular defects were classified using the Paprosky classification. Large defects were seen in 42 cases (type 3A: 20, type 3B: 21, type 4: 1). Furthermore, ten moderate defects were found according to type 2 (type 2B: 5, type 2C: 5). There were no smaller defect sizes in our evaluation. The system was used 11 × for periprosthetic acetabular fractures with inserted HEP, 5 × for acetabular loosening with inserted HEP, 4 × for dislocation with inserted HEP, 4 × for periprosthetic femoral fracture and loosened acetabulum, 3 × in acetabular metastasis with underlying malignancy, 1 × in marked acetabular deformity with severe coxarthrosis, 16 × in primary acetabular fracture in native femoral head, 1 × in acetabular fracture after DHS instrumentation, 3 × in secondary in acetabular re-fracture after plate osteosynthesis and 4 × in acetabular substance defect after hip joint infection.

In 19 cases, only the cup was replaced and in 33 cases the cup with the stem. Bone augmentation was performed using autologous cancellous bone in 15 cases, allogeneic cancellous bone 16 times, and no cancellous bone was needed in 21 cases. On average, 4.2 fixation screws were used (2–7 screws). Furthermore, the procedure of the operation was analyzed. The effective operation time was 02:36 h (01:06–05:06 h) and the intraoperative blood loss was 1313 ml (100–3500 ml). A mechanical autotransfusion was used 22 times, from which a reinfused volume of 225 ml (26–1077 ml) could be returned to the patient. Nine patients received preoperative transfusion with an average of two red cell concentrates, intraoperative transfusion requirement was one red cell concentrate (0–6 units). In the postoperative monitoring period, transfusions by red cell concentrate were 1.7 units (0–7 units). Considering the transfusion needs in the whole inpatient stay, the required red cell concentrates increased to 4.2 units (0–30 units). The intraoperatively inserted drains pumped a volume of 1125 ml (30–3340 ml) until removal. Intraoperatively, there were moderate, manageable surgical complications. With very poor bone quality, 9 × cement fixation of the screw heads and 1 × composite osteosynthesis were performed. Due to a contracted overall situation, 3 × a step osteotomy was surgically corrected due to ossification and soft tissue contracture. 1 × the decision for a MUTARS^®^ RS Cup implantation was made intraoperatively, because the ventral part of the acetabulum was unstable when reaming the cup. In two patients, hemorrhagic shock with extended postoperative ventilation was observed.

Furthermore, surgical complications appeared postoperatively. Four cases were revised for head luxations, twice the acetabular component was changed and twice a tripolar cup was inserted into the existing MUTARS^®^ RS Cup. Moreover, in one case an arterial rebleeding with necessity of coil embolization by the colleagues of the radiology department without further surgical measures was detected. Deep postoperative wound infection needed to be surgically sanitized five times.

Neither acetabular protrusion nor acetabular perioperative fracture were observed. Modular prosthesis disengagement or component breakage has not been observed in these patients, as well an inlay dislocations were not observed. The radiological assessment of the postoperative X-rays was analyzed in all revised cases, with the postoperative radiograph at baseline being compared with controls at subsequent follow-up examinations. At the last follow-up in the patients, there loosening of the acetabular titanium cup was not noted. All implanted cups showed signs of stability in the X-ray. Most often, the MUTARS^®^ RS Cup with the size 54 mm used. Furthermore, the common stems used in our hospital were used for the respective indication and a Polyethylen neutral inlay was chosen most frequently (Table 1).

**Table 1:** Table with the used implants for the cup, stem and inlay

Size MUTARS^®^ RS cup	46 mm	50 mm	54 mm	58 mm	62 mm
Use	2×	14×	22×	14×	0×
Stem	MS-30^®^	Revitan^®^	CLS^®^	IC^®^ long	old stem
Use	17×	9×	6×	1×	1×
Inlay	Polyethylen neutral 0 mm	Polyethylen offset 4 mm	EcoFit 2 M^®^		
Use	32×	9×	11×		

## Discussion

Acetabular revision surgery is challenging due to the occurrence of bony defects that hinder primary fixation of implants when removing loosened components. Reinforcement rings, such as Burch-Schneider (BS) rings, have proven to be a good possibility in moderate to severe bony defects over many decades without substantial changes.

The BS reinforcement ring showed moderate and varying results in the medium and long term, allowing both anatomical reconstruction in revision surgery and replacement of the bone stock [13]. However, there are still challenges associated with its use. The cup has to be cemented into the revision ring to achieve a stable anchorage, and this is usually a low profile polyethylene cup. Furthermore, the extension of a tripolar system cannot be anchored in the BS ring, a concept that has now been established as the best option for repeated luxation in instable hip arthroplasty [18]. The patient clientele has also changed over time. We are increasingly confronted with very old patients, frequent and repeated revision surgeries with inserted prostheses and also increased osteoporosis. Therefore, there are more indications for secure anchorage and implantation of more complex systems and the greater need for a next level system. The mean age of patients was 76,1 years (36,9–94,4 years), 28 male and 21 female patients. In addition, large defects of the acetabulum have been noted in basically all cases. 4/5 of the cases were classified in the highest Paprosky classification.

Further development has created the MUTARS^®^ RS Cup prosthesis, which was applied in our department. Here, we have an integrated inlay that does not need to be cemented in, thus similar to any standard titanium prosthesis. Furthermore, a larger head can be inserted, which significantly increases stability. Also, there is the possibility to insert a bipolar cup- leading to a tripolar system [18], which is getting the increasing standard in complex revision surgery. In our cases, we have therefore used the option of tripolar prosthesis in 11 cases, mostly after luxation or instable situations. The MUTARS^®^ RS cup system is well-designed system that in many cases allows a safe reconstruction of the acetabulum. All, acetabular fractures and post-traumatic osteoarthritis, as well as non-inflammatory degenerative joint disease, including osteoarthritis and avascular necrosis, might be indications to use the MUTARS^®^ RS cup system.

In addition, revision hip arthroplasty of pans of all kinds are possible, whereas in former times only the whole cup or a Burch-Schneider ring existed. Even large cavitary or segmental acetabular defects (up to type IIIa/IIIb of Paprosky classification) can be treated with the system. Another widespread application is trauma in elderly patients, especially in cases where a normal cup, even when cemented, is not attachable. For the reconstruction of bone defects, the application of allogenic bone grafts may be an option when using the MUTARS^®^ RS cup [17]. However, there are also limitations when inserting the MUTARS^®^ RS. If the acetabulum is too unstable, e.g., in pelvic ring fractures with participation of the acetabulum, the cup gets not enough fixation and can protrude into the small pelvis. In this case, bone grafts become necessary more often or the additional stabilization of the pelvic ring is warranted.

There are some relative contraindications to be considered. Anatomical conditions, especially, must be considered and in particular insufficient possibility of fixation into an unstable pelvic ring needs to be considered, and the pelvic ring has to be stabilized separately in advance. If the support of the implant is not given or the implantation of a sufficiently large denture is not possible, it should not be used. Insufficient quantity and quality of the bone stock, e.g., due to osteoporosis or osteomalacia, may also make a safe implantation impossible. In addition, diagnosed vascular diseases of the affected extremity must be taken into account, as well as metabolic disorders that can impair stable implantation. Bone tumors in the area of the implant fixation or neuromuscular diseases that may affect the affected extremity may also be contraindications.

The MUTARS^®^ RS system as an innovative development of the combination of a Burch-Schneider ring and a cemented polyethylene cup, combines several advantages in one system. There is no need to cement the acetabulum, which eliminates the risk of intraoperative cement embolisms, cement protrusion into the pelvis or cement loosening. The material used is the widely used titanium, which is well tolerated and stable. The back of the MUTARS^®^ RS prosthesis highly porous EPORE^®^ structure is designed to strengthen the biological ingrowth of bone. In addition, the prosthesis has two wings which allow fixation in the ramus ossis ischii by means of the tongue and in the cranial acetabulum in the corpus ossis ilii by means of screws, thus the basis of the Burch Schneider ring concept [17]. Furthermore, it is possible to latch the inlay to the system, which improves the anchoring, eliminates the need for cement/cup loosening and reduces the tendency of the inlay to dislocate. They can be used with a raised edge, which additionally improves the prevention of luxation. In addition, a larger head of the prosthesis can be used with the clicked-in inlay, which also reduces the tendency to dislocation. In case of a pronounced acetabular defect, a cup base construction is possible. The system offers the option of a bipolar prosthesis.

## Conclusion

**Fig. 4:**
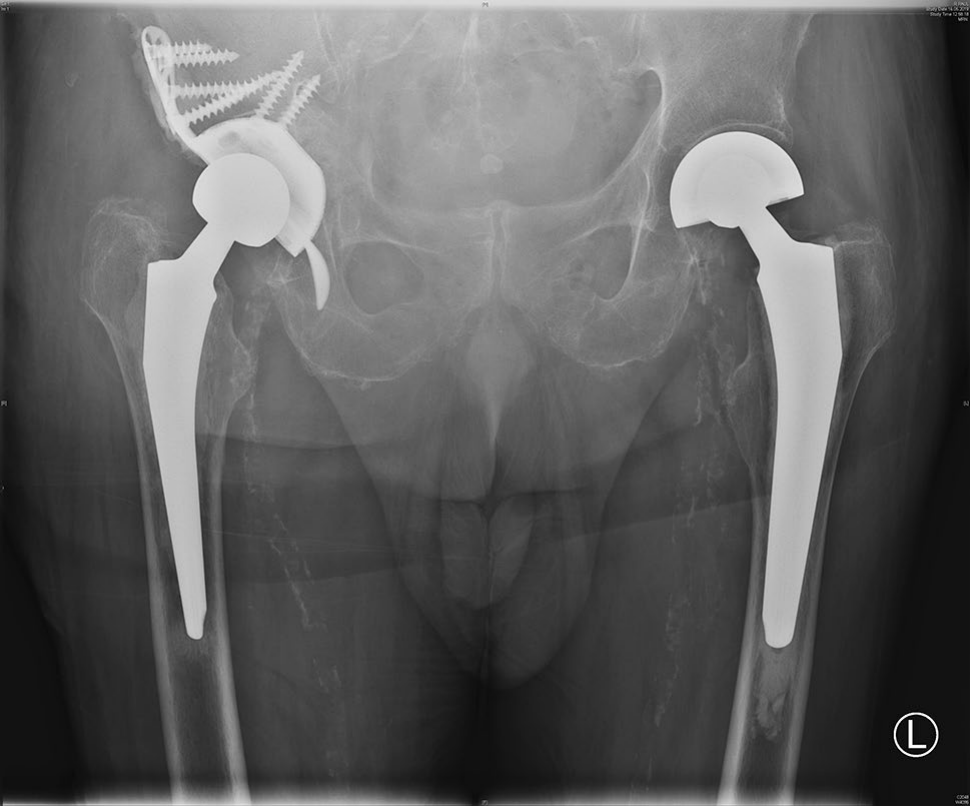
Well-fitting MUTARS^®^ RS cup at the right hip in the pelvis overview

The use of an acetabular cup reconstruction system with an integrated cup fixation, even if there are cavitary and segmental bone defects in revision operations is a valuable tool for critical acetabular fractures and defects. This is a substantial improvement over the cementation of a polyethylene cup into a revision cup, such as the Burch Schneider ring. The modularity of the applied MUTARS^®^ RS CUP system makes it possible to correct the inlay position, the center of hip rotation and to minimize the risk of dislocation [19, 20] (Fig. 4). Additionally, many optional constructs are available to achieve sufficient acetabular cup-bone contact and return the hip center to normal anatomical position. Moreover, the use of bone allografts or porous acetabular metal enlargements is possible. Clinical evaluation of the MUTARS^®^ RS Cup revealed good results in terms of clinical performance and safety in this population of complicated revision situations and acetabular fractures, mostly in older or previously operated patients. Although, we also observed the requirement for revisions is certain cases, it must be taken into account that this is an extensive operation with an often complicated, even unique, anatomical situation in this critical patient population. The stabilization of the acetabular bone defects at the time of revision of the THA is the key factor for a successful outcome in hip revision surgery. Based on the results of this evaluation of the MUTARS^®^ RS Cup, the use of an integrated cup in a Burch-Schneider ring with various intraoperative options may be useful to solve complex acetabular situations, periprosthetic acetabular fractures or revision arthroplasty.
